# Social Notes

**Published:** 1920-11-20

**Authors:** 


### Social Notes.

By A SOCIAL WORKER.

Nurses and other workers who have seen service in France often wish they could "keep up their French" and also learn more about the country in which they became so much interested. The University of Lille, in the heart of the devastated area, has now "adopted" London as that of Toulouse formerly adopted Petrograd. Grenoble has also made an adoption. This means that in London a branch of Lille now works with the local University in a fine building—3-7 Cromwell Gardens, facing the Victoria and Albert Museum. Here, besides regular schools for children, there are classes in preparation for the French *baccalauréat* and the English matriculation, and for teachers and others. There are also most attractive courses of lectures on French music, French classics, the life of Bonaparte, and some phases of French social life. Single, these cost half-a-crown; term subscription, £3 3s. But there is also the generous gift of a series of *free* lectures in French from November 6 to December 18, on Thursdays at 6.15, and on Saturdays at 3, all on attractive subjects. Such chances are rare.

Those of us who have stopped during a walk in one of the parks to listen to a platform speaker who stands for the faith of Christians may have wondered where he came from. The Christian Evidence Society has quietly worked to provide these valiant speakers; some of whom are working men, and sends them also to be present at Rationalist and Bolshevist meetings, where they have done much in upholding truth and loyalty in religion and civil life. They are also arranging meetings for teachers and others, and will give help to those who want to know how to help others. Many a nurse has to confront the troubled mind of her patient in night watches or times of sinking. Some may like to know of the devotional meeting now held on Sundays from 6 P.M. to 6.55 P.M. at 2 Cumberland Mews, a turning on the right-hand side of Edgware Road going north, a few yards only from Marble Arch. The Society's office is at 34 Crabbe Street, Strand. Secretary, the Rev. C. L. Drawbridge.

